# Protein-Bound Polysaccharide from *Corbicula fluminea* Inhibits Cell Growth in MCF-7 and MDA-MB-231 Human Breast Cancer Cells

**DOI:** 10.1371/journal.pone.0167889

**Published:** 2016-12-13

**Authors:** Ningbo Liao, Jianjun Zhong, Ronghua Zhang, Xingqian Ye, Yanjun Zhang, Wenjun Wang, Yuexia Wang, Shiguo Chen, Donghong Liu, Ruihai Liu

**Affiliations:** 1 Department of Nutrition and Food Safety, Zhejiang Provincial Center for Disease Control and Prevention, Hangzhou, Zhejiang, China; 2 College of Biosystem Engineering and Food Science, Zhejiang University, Hangzhou, Zhejiang, China; 3 Zhejiang Academy of Science & Technology for Inspection & Quarantine, Hangzhou, Zhejiang, China; 4 Fuli Institute of Food Science, Zhejiang University, Hangzhou, Zhejiang, China; 5 Department of Food Science, Cornell University, Ithaca, New York, United States of America; University of the Witwatersrand, SOUTH AFRICA

## Abstract

A novel protein-bound polysaccharide, CFPS-1, isolated from *Corbicula fluminea*, is composed predominantly of mannose (Man) and glucose (Glc) in a molar ratio of 3.1:12.7. The polysaccharide, with an average molecular weight of about 283 kDa, also contains 10.8% protein. Atomic force microscopy, high-performance liquid chromatography, Fourier transform infrared spectroscopy, gas chromatography/mass spectrometry, and nuclear magnetic resonance spectroscopy analyses revealed that CFPS-1 has a backbone of 1,6-linked and 1,4,6-linked-α-D-Glc, which is terminated with a 1-linked-α-D-Man residue at the O-4 position of 1,4,6-linked-α-D-Glc, in a molar ratio of 3:1:1. Preliminary *in vitro* bioactivity tests revealed that CFPS-1 effectively and dose-dependently inhibits human breast cancer MCF-7 and MDA-MB-231 cell growth, with an IC_50_ of 243 ± 6.79 and 1142 ± 14.84 μg/mL, respectively. In MCF-7, CFPS-1 produced a significant up-regulation of p53, p21, Bax and cleaved caspase-7 and down-regulation of Cdk4, cyclin D1, Bcl-2 and caspase-7. These effects resulted in cell cycle blockade at the S-phase and apoptosis induction. In contrast, in MDA-MB-231, with limited degree of change in cell cycle distribution, CFPS-1 increases the proportion of cells in apoptotic sub-G1 phase executed by down-regulation of Bcl-2 and caspase-7 and up-regulation of Bax and cleaved caspase-7. This study extends our understanding of the anticancer mechanism of *C*. *fluminea* protein-bound polysaccharide.

## Introduction

The freshwater clam *Corbicula fluminea* is a popular edible bivalve mollusk in Asia. It has been important in the human diet since ancient times in China because of its delicious taste and nutritional value [1]. We previously reported that a *C*. *fluminea* sulfate polysaccharide, CFPS-2, displays marked inhibitory effects on the growth of SKOV3 human ovarian carcinoma cells and SGC7901 human gastric cancer cells [2]. Zhu et al. reported that another bioactive glycoprotein, CFp-a, from *C*. *fluminea* exerted antitumor activity on BEL7404 cells by inducing their apoptosis [3]. Several studies have recently reported that extracts of *C*. *fluminea* have a broad range of biological properties, including hepatoprotective [4], antioxidant [5], anticancer [6], antihypertensive [7], and hypocholesterolemic activities [8]. However, the active constituents of *C*. *fluminea* have not been studied in detail.

Breast cancer is the most common malignant disease among women, and approximately one-third of women in the world with breast cancer develop metastases and die [9]. Although many tumors respond initially to chemotherapy, breast cancer cells can become resistant to treatment and therefore survive. Thus, searching for new alternative breast cancer treatments is necessary. *In vitro* experiments have shown that protein-bound polysaccharides prepared from natural sources (e.g., fungi, plant, algae, animals, and bacteria) exert anticancer activities on many kinds of cancer cells, including breast, prostate, lung, stomach, and lymphoma cancer cell lines [10]. Kidd et al. reported that in double-blind trials, a protein-bound polysaccharide from a mushroom significantly extended the survival of patients with esophageal adenocarcinoma [11]. However, the exact mechanisms underlying the direct inhibitory effects of protein-bound polysaccharides on cancer cell growth are not well understood.

In this study, a novel polysaccharide–protein complex (designated ‘CFPS-1’) was extracted from *C*. *fluminea* and purified. Its molecular characteristics, including its morphology, molecular weight (Mw), and chemical structure, were determined with atomic force microscopy (AFM), high-performance liquid chromatography (HPLC), nuclear magnetic resonance (NMR), Fourier transform infrared spectroscopy (FT-IR), and gas chromatography/mass spectrometry (GC/MS). We also investigated the anticancer activity of CFPS-1 against human breast cancer MCF-7 and MDA-MB-231 cells and their possible inhibitory mechanisms.

## Materials and Methods

### Materials and Reagents

*Corbicula fluminea* was purchased from Fenren Foodstuff Co., Ltd, Hangzhou, China. Ethylenediaminetetraacetic acid (EDTA), sodium dodecyl sulfate (SDS), nonfat milk powder, and bovine serum albumin were obtained from Sigma-Aldrich Chemical Co. (St. Louis, MO, USA). The disaccharide lactose, monosaccharide standards, 1-phenyl-3-methyl-5-pyrazolone (PMP), papain, and cysteine were from Sinopharm Chemical Reagents Co., Ltd (Shanghai, China). Rabbit polyclonal anti-Bax antibody and rabbit polyclonal anti-caspase-7 antibody were from Santa Cruz Biotechnology, Inc. (Santa Cruz, CA, USA). Mouse monoclonal antibodies directed against human p53, p21, Cdk4, cyclin D1, and Bcl-2 were obtained from Abcam (Cambridge, MA, USA). A horseradish peroxidase (HRP)-linked anti-mouse IgG secondary antibody was obtained from Cell Signaling Technology (Danvers, MA, USA). Other reagents used in this study were all of analytical grade.

### Extraction and Chemical Analysis of CFPS-1

The procedures used to isolate and extract CFPS-1 have been described in detail in our previous paper [2]. Purified CFPS-1 isolated from *C*. *fluminea* is a white powder, with a yield of 0.93% of the dry material after lyophilization. The relevant data have been reported previously [2]. The carbohydrate content was analyzed with the phenol–sulfuric acid method [12]. The molecular weight and homogeneity of CFPS-1 were determined on a Waters Alliance 2695 HPLC system equipped with a differential refractometer (Waters 2410, Millipore, Milford, USA). The monosaccharide composition of CFPS-1 was determined with the HPLC method used for PMP derivatization [13]. The sulfate content was determined with ion-exchange chromatography and the BaCl_2_ gelatin method [14]. The protein concentration and amino acid constituents were determined with the Lowry method and HPLC AccQ method, respectively [15,16]. The tryptophan content was quantified with alkaline hydrolysis and UV detection at 280 nm.

### Atomic Force Microscopy (AFM)

Images of CFPS-1 were obtained with AFM, as reported previously [17]. The original CFPS-1 solution (10 mg/mL) was diluted with double-distilled water to final concentrations of 100, 10, and 1 μg/mL. A drop of butanol was added to each diluted CFPS-1 solution, and about 5 μL of diluted solution was spread onto a freshly cleaved mica surface, air-dried overnight, and imaged at room temperature. Tapping mode images were taken with a Nanoscope III digital instrument (Santa Barbara, CA, USA), with all the steps performed at 30%–40% relative humidity and ambient air pressure.

### Methylation Analyses

The polysaccharide fraction of CFPS-1 (150 mg) was methylated three times with a previously reported method [18]. The per-O-methylated product showed no OH absorption (3600–3300 cm^–1^) in the IR spectrum, and was then hydrolyzed with 90% formic acid (HCOOH) for 15 h at 100°C. The hydrolyzed product was concentrated to dryness, and the partially methylated residues were reduced and acetylated. The partially O-methylated alditol acetates were analyzed qualitatively and quantitatively with a GC/MS apparatus (Thermo Finnigan, CA, USA) fitted with an OV1701 capillary column (30m × 0.25 mm internal diameter). The column was first held at 150°C during injection and then programmed to increase to 250°C at a rate of 3°C/min. The methylated sugar linkages were identified by their typical electron impact spectra and relative retention times.

### FI-IR and NMR Analyses

The CFPS-1 sample (2 mg) was pressed into a KBr pellet at room temperature. Infrared spectroscopy was performed within the range of 400–4000 cm^−1^ on a Nicolet 5700 FT-IR spectrometer (Thermo Electron, Madison, WI, USA) and analyzed with OMNIC 7.0 software. For the NMR measurements, CFPS-1 was dissolved in D2O to exchange its active hydrogen and was then lyophilized. This procedure was repeated several times. In total, 40–50 mg of deuterium-exchanged CFPS-1 sample was dissolved in 0.5 mL of D_2_O in an NMR tube to detect the NMR spectra. The NMR spectra (^1^H NMR and ^13^C NMR) were recorded on a Bruker Avance^™^ 600 spectrometer (600 MHz) at 60°C.

### Cell Lines and Cell Cultures

The human breast cancer cell lines MCF-7 and MDA-MB-231 were purchased from the American Type Culture Collection (Rockville, MD, USA) and grown in minimum essential medium supplemented 10% fetal bovine serum (for MCF-7) or 10% heat-inactivated fetal bovine serum (for MDA-MB-231), 10 mM HEPES, 50 units/mL penicillin, 100 μg/mL gentamicin, 50 μg/mL streptomycin, and 10 μg/mL insulin.

### MTS Assay

The cell numbers were quantitated with 3-(4,5-dimethylthiazol-2-yl)-5- (3-carboxymethoxyphenyl) -2 - (4-sulphophenyl)-2H-tetrazolium (MTS) assay [19]. The MCF-7 and MDA-MB-231 cells were incubated at 37°C with 5% CO_2_ in air. To assess the effects of CFPS-1 on the growth of the breast cancer cell lines, MCF-7 and MDA-MB-231 cells (2.5 × 10^4^ cells/well) were seeded in 96-well flat-bottom plates. After 4 h in culture, the cells were treated with different concentrations of CFPS-1 (0, 50, 150, or 250 μg/mL) for 24, 48, 72, or 96 h. The breast cancer cells were incubated with MTS dye (2 mg/mL) for 2 h (20 μL/well) and solubilized with 25 ml of 10% sodium dodecyl sulphate (SDS) at room temperature for 4 h. Absorbance was determined in a MRX II Dynex plate reader at 490 nm. The absorbance is directly related to viable cell number. Cell viability was expressed as the ratio of the mean absorbance of treated cells to that of control cells. Based on these results, breast cancer cells treated with CFPS-1 for 72 h were selected for subsequent experiments.

### Cytotoxicity and Proliferation Inhibition Assays

Two assays were used to evaluate the cytotoxicity and antiproliferative effects of CFPS-1, in which CFPS-1 treatment was applied for 24 h and 72 h, respectively [20]. To avoid the cultures becoming confluent during the CFPS-1 treatments, adequate quantities of exponentially proliferating cells were used to evaluate the cytotoxic effects (4.0 × 10^4^ cells/well) and possible antiproliferative effects (2.5 × 10^4^ cells/well) of CFPS-1 in 96-well flat-bottom plates. CFPS-1 was added to the wells 24 h after seeding (to maximize the environmental adaptation of the cells) for 24 or 72 h and then removed. The cultures were then maintained for another 24 h before cell viability was quantified. The concentrations of CFPS-1 used were 93, 187, 375, 750, and 1500 μg/mL, with the control groups receiving the extract solution without the extract. Cytotoxicity and proliferation were determined with MTS assay as described above [19], and expressed as follows [21]:

$$Cytotoxicity\;\left(\%\right)=\left(1-\;\frac{Absorbance\;of\;treated\;cells}{Absorbance\;of\;control}\right)\times 100.$$ (1)

$$Proliferation\;\left(\%\right)=\frac{Absorbance\;of\;treated\;cells}{Absorbance\;of\;control}\times 100.$$ (2)

A minimum of three replications for each sample was used to determine the cells cytotoxicity and proliferation. Inhibitory concentrations of 50% (IC50) for CFPS-1 were calculated using Calcusyn software (Biosoft, Ferguson, MO, USA).

### 5-Bromo-2-deoxyuridine (BrdU) Assay

The effects of CFPS-1 on DNA synthesis in proliferating cells were assessed by BrdU assay, with a previously reported method [22]. Briefly, the cells were incubated with increasing concentrations of CFPS-1 for 96 h in normal culture medium at 37°C in a 96-well cell culture plate in a 5% CO_2_ humidified incubator. BrdU reagent was added to each well and incubated for 16 h. The medium was removed and BrdU incorporation was measured according to manufacturer’s instructions (Roche, Mannheim, Germany). The color intensity in each well was measured at a wavelength of 450 nm.

### Determination of Cell-Cycle Phase Distribution

MCF-7 and MDA-MB-231 cells were seeded in 12-well flat-bottom plate at a density of 1 × 10 ^6^ cells/well, and cultured to reach 60%–70% confluence after 6 h. They were then treated with different doses of CFPS-1 (0, 50, 150, or 250 μg/mL) for 24 h. After treatment, the cells were harvested by trypsinization, washed in PBS, and fixed in 70% ethanol. The fixed cells were resuspended in 1 mL of PBS containing 50 mg/mL propidium iodide and 1 mg/ml RNase for at least 30 min. The mixture was then analyzed with flow cytometry (BD FACSVerse^™^, BD Biosciences, Franklin Lakes, NJ, USA), and the data were analyzed with the ModFit LT ver. 2.0 software [22]. The sub-G1 cell fraction was considered to represent apoptotic cells.

### Determination of Cell Apoptosis

The induction of apoptosis by CFPS-1 was assessed *in vitro* with a terminal-deoxynucleotidyl-transferase-mediated dUTP nick end-labeling (TUNEL) assay [23]. MCF-7 and MDA-MB-231 cells were placed in a Falcon eight-chamber culture slide at a density of 3 × 10^5^ cells/well, allowed to grow to 60%–70% confluence, and then serum-starved overnight in medium without FBS. The starved cells were exposed to different doses of CFPS-1 (0, 50, 150, or 250 μg/mL) for 24 h and then collected for the analysis of apoptosis with an ApopTag In Situ Apoptosis Detection Kit (Serologicals Corporation, Norcross, GA, USA), according to the manufacturer’s instructions. Cell apoptosis was assessed with light microscopy (magnification, × 40).

### Protein Extraction and Western Blotting Analysis

MCF-7 and MDA-MB-231 cells were seeded in six-well flat-bottom plates, and grown for 24 h. The cells were then exposed to different concentrations of CFPS-1 (0, 50, 150, or 250 μg/mL) for 24 h. After incubation, the cells were washed once with ice-cold PBS and then lysed in lysis buffer (1% Igepal CA-630, 1 mM EDTA, 150 mM sodium chloride, 50 mM Tris [pH 7.4]) containing protease inhibitors (1 μg/mL leupeptin, 1 μg/mL aprotinin, 1 mM phenylmethanesulfonyl fluoride, 1 mM sodium fluoride, 1 mM sodium orthovanadate, 1 μg/mL pepstatin) for 20 min on ice with occasional vortexing. The protein concentration in the lysate was measured with the Total Protein Determination Kit (Sigma-Aldrich). Equal amounts of protein (15 μg) were separated with SDS-polyacrylamide gel (12%) electrophoresis and transferred to Immobilon-P membranes (Millipore, Billerica, MA, USA). The membranes were blocked with 3% nonfat dried milk in PBS, and incubated overnight with primary antibody at 4°C. The membrane was rinsed three times and incubated with the corresponding HRP-conjugated secondary antibody in PBS with 3% nonfat milk powder for 1 h at room temperature with agitation. The protein bands were visualized with the Phototope-HRP Western Blot Detection System (Cell Signaling, Danvers, MA, USA) and analyzed with the UVP Labworks software (Upland, CA, USA). β-Actin was used as the internal control.

### Statistical Analysis

Data are given as means ± standard deviations. The unpaired Student’s t test was used to compare two means. The differences between more than two means were evaluated with analysis of variance followed by Fisher’s least significant difference test for multiple comparisons. Differences were considered significant at p ≤ 0.05.

## Results

### Imaging and Composition Analysis of CFPS-1

**Fig 1:**
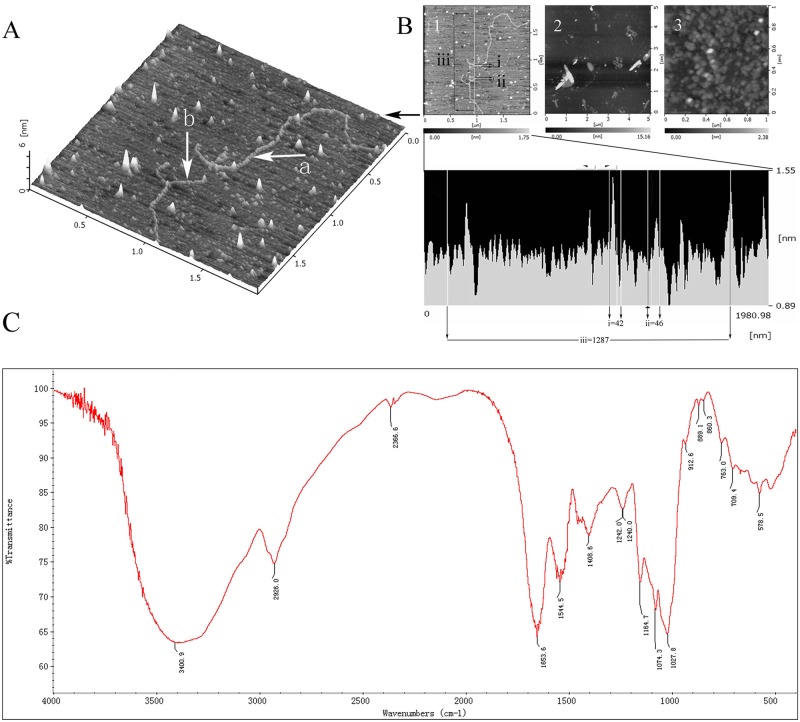
Molecular and morphological characterization of *C*. *fluminea* protein-bound polysaccharide CFPS-1. (A and B) The atomic force microscopy (AFM) images of CFPS-1. The CFPS-1 concentration was 1 μg/mL (A; B1), 10 μg/mL (B2) and 100 μg/mL (B3), respectively. Arrow: (a) single linear chain; (b) branched chain; (C) FTIR spectra of CFPS-1 (4000 cm^−1^ to 400 cm^−1^) were obtained from solid samples by KBr disc method using a Nicolet 5700 FT-IR spectrophotometer. FTIR spectra showed that CFPS-1 mainly contained three types of groups, namely hydroxyl group, amino group and sulfate group.

The purified CFPS-1 isolated from *C*. *fluminea* was a white powder, with a yield of 0.93% of the dry material after lyophilization. In our previous study, the high-performance size exclusion chromatography profile of CFPS-1 showed as a single peak, corresponding to a mean *M*w of 283 kDa [2]. The composition and structure of CFPS-1 was further clarified in this study. As shown in Fig 1, the profiles of the CFPS-1 molecule in solution at different concentrations (1.0–100.0 μg/mL) were observed with AFM. Fig 1A clearly shows a fiber-like structure with small branches of the CFPS-1 molecule in the low-concentration solution (1 μg/mL). The length of the molecule was approximately 1100–1300 nm and the height was 42–46 nm. When the concentration was high (100 μg/mL), the CFPS-1 molecule formed a strong three-dimensional ball-like structure (Fig 1B; 100 μg/mL). The total carbohydrate content of CFPS-1 was 82.3% when measured with the phenol–sulfuric acid method. Analysis of the monosaccharide components of CFPS-1 showed that it is mainly composed of mannose (Man) and glucose (Glc) in a molar ratio of 3.1:12.7. Galactose (Gal), fucose (Fuc), glucuronic acid (GlcUA), and glucosamine (GlcN) were also detected in minor amounts. The sulfate content of CFPS-1 is 2.2%, and a small amount of protein (10.8%) was also detected (Table 1). An amino acid analysis of the protein fraction of CFPS-1 is shown in Table 2. Fifteen amino acids were detected in the polymer, and essential amino acids constituted 25% of the total amino acids. Glutamic acid was the main amino acid (40.9 mg/g), followed by serine (8.4 mg/g) and threonine (7.28 mg/g). The results shown in Tables 1 and 2 indicate that CFPS-1 is probably a protein-bound polysaccharide.

**Table 1:** Yield, chemical compositions and contents of carbohydrate, protein, *M*_W_ and sulfonic acid in the *C*. *fluminea* protein-bound polysaccharide CFPS-1.

Item	Content ^a^
Yield (%) ^b^	0.9 ± 0.3
Protein (%)	10.8 ± 2.7
Neutral sugar (%)	82.3 ± 6.7
Sulfonic acid (%)	2.2 ± 0.8
*M*_W_(kDa) ^c^	283 ± 7.0
Molar ratio of monosaccharides ^d^	
Man	3.07
GlcN	0.28
Glu	12.73
Gal	0.57
Fuc	0.55

^a^ Results are presented as mean ± SD (n = 3).

^b^ Data are expressed in g/100g dry weight

^c^ Molecular weight

^d^ Abbreviation is as follows: Man, Mannose; GlcN, glucosamine; Glc, glucose; GalN, galactosamine; Gal, galactose; Fuc, fucose.

**Table 2:** The amino acid composition of the protein-bound polysaccharide CFPS-1 from *C*. *fluminea*.

Amino acid ^c^	Concentration (mg/g) ^a^
Asp	4.74 ± 1.21
Ser	8.39 ± 1.35
Glu	40.87 ± 2.22
Gly	0.39 ± 0.12
His	4.45 ± 0.55
Arg	6.12 ± 1.61
Thr ^b^	7.28 ± 1.68
Ala	3.34 ± 1.68
Pro	3.12 ± 1.34
Cys_2_	4.23 ± 0.26
Tyr	0.18 ± 0.13
Val ^b^	6.37 ± 0.56
Met ^b^	6.34 ± 1.44
Lys ^b^	4.32 ± 0.36
Leu ^b^	1.19 ± 0.28
Total amino acids	101.33 ± 13.75
Proportion of the essential amino acids (%)	25.17 ± 3.32

^a^ Data were shown as mean ± SD (n = 3)

^b^ Essential amino acids

^c^ Abbreviation is as follows: Asp, Asparagine; Ser, Serine; Glu, Glutamic acid; Gly, Glycine; His, Histidine; Arg, Arginine; Thr, Threonine; Ala, Alanine; Pro, Proline; Cys_,_ Cysteine; Tyr, Tyrosine; Val, Valine; Met, Methionine; Lys, Lysine; Leu, Leucine

### UV and IR Spectra of CFPS-1

Absorption was observed at 280 nm on the UV spectrum of CFPS-1, suggesting the presence of protein in CFPS-1. The IR spectrum of CFPS-1 suggested that the main absorption bands in Fig 1C are attributable to the stretching vibrations of O–H (3401 cm^–1^), C–O (1028 cm^–1^), C–O–C (1165 cm^–1^), and an anomeric C_1_H group (1074 cm^–1^). The strong band at 1242 cm^-1^ and the weak band at 850 cm^–1^ are associated with the stretching vibrations of C–O–S and S–O, respectively, indicating the presence of a sulfate ester [24, 25]. The characteristic absorption at 1654, 1544, and 1409 cm^–1^ is attributed to the presence of protein in CFPS-1 [26], which are similar to those from the UV spectrum. However, the stretching vibration of N–H (3400 cm^–1^) could overlap the O–H stretching vibration at 3410 cm^-1^. The protein in CFPS-1 is also represented by the band at 1242 cm^–1^. Purified CFPS-1 also displays α-glycosidic bonds at 854 cm^–1^. All these findings suggest that the polysaccharide CFPS-1, containing α-glycosidic bonds, is a proteoheteroglycan.

### Structural Data from Methylation Analysis

Methylated CFPS-1 was hydrolyzed to form alditol acetates and then analyzed with GC/MS. Table 3 shows the presence of three major compounds, 2,3,4- Me_3_-Glc, 2,3,- Me_2_-Glc, and 2,3,4,6- Me_4_-Man, which indicate the presence of 6-linked Glc, 4,6-linked Glc, and terminal Man, in a molar ratio of 3:1:1. The high proportion of 6-linked Glc indicates that the main consecutive repeating unit of CFPS-1 is (1→6)-linked Glc. The equal proportions of terminal Man and 4, 6-linked Glc indicate the presence of a branched Man attached to the 4-O position of Glc in the backbone of CFPS-1.

**Table 3:** GC-MS of alditol acetate derivatives from the methylated product of the *C*. *fluminea* protein-bound polysaccharide CFPS-1.

Methylated sugar	Molar ratio	Mass fragment (*m/z*) ^b^	Type of linkage
2,3,4-Tri-*O*-Me-Glc ^a^	3.24	43, 45, 71, 87, 101, 117, 129, 161, 173, 189, 233	→6)-Glc*p*-(1→
2,3,-Di-*O*-Me-Glc	1.3	43, 56, 85, 99,127,141, 159, 201, 261	→4, 6)-Glcp-(1→
2,3,4,6-Tetra-*O*-Me-Man	1.08	28, 43, 71, 87, 101, 117, 129, 145, 161,205	Man*p*-(1→

^a^ 2,3,4-Tri-*O*-Me-Glc = 1,5,6-tri-*O*-acetyl-2,3,4-tri-*O*-methyl-D-glucitol.

^b^ Equipped with a OV1701 capillary column (30m×0.25mm internal diameter) using a temperature program from 150°C (2 min) to 250°C (5 min) at 3°C min^-1^.

### Structural Data from NMR

**Fig 2:**
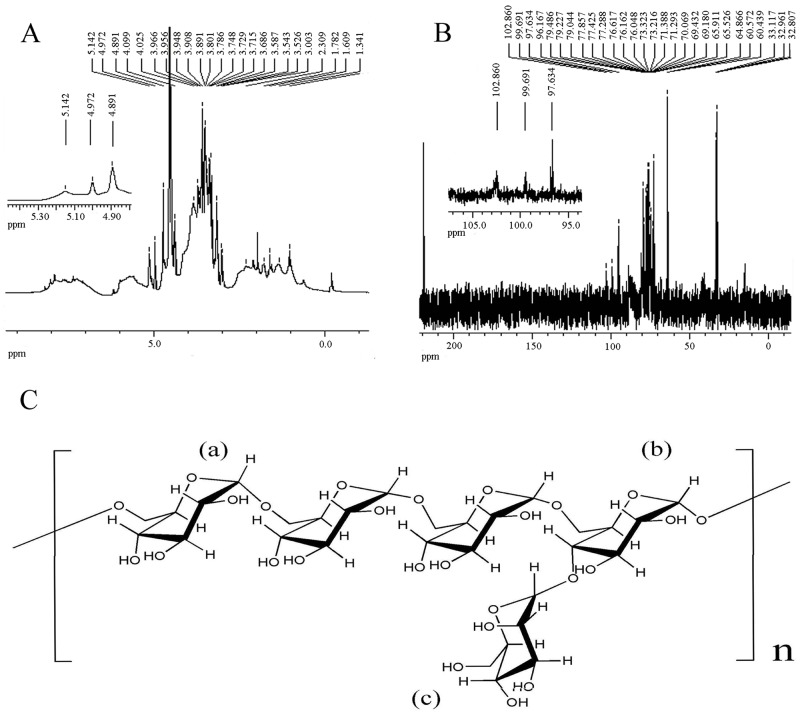
Structural elucidation of the *C*. *fluminea* protein-bound polysaccharide CFPS-1. **(A)** ^**1**^**H NMR and (B)** ^**13**^**C NMR spectra of CFPS-1 in D**_**2**_**O.** Chemical shifts were reported relative to internal acetone at δH 2.23 ppm for ^1^H spectrum and δC 32.45 ppm for ^13^C spectrum; (C) A diagram showing the partial structures of polysaccharide portions of the protein-bound polysaccharide CFPS-1 from C. fluminea. (a): 1,6-linked -α-D-Glc; (b): 1,4,6-linked-α-D-Glc; (c): 1-linked-α-D-Man.

The analysis of the NMR spectra (1H NMR and 13C NMR) is supported by information derived from the literature, the constituent analysis, and the linkage patterns of the molecule (Table 4). The ^1^H NMR spectrum of CFPS-1 is shown in Fig 2A. The chemical shifts at δ 4.89, δ 4.98, and δ 5.14 ppm imply that the sugar rings of CFPS-1 are α-type pyranose. The signal in the region δ 3.0–4.3 ppm is attributed to C2–C6 protons, and the characteristic signals in the regions δ 0.6–3.1 and δ 5.5–6.4 ppm correspond to amino groups and sulfated groups, respectively [27,28]. In the ^13^C NMR spectrum of CFPS-1 (Fig 2B), the main α-Glc (1→6) residues are characterized by six obvious resonance signals in the regions δ 69–98 ppm for C1 (δ 97.63 ppm), C2 (δ 71.31 ppm), C3 (δ 73.27 ppm), C4 (δ 70.09 ppm), C5 (δ 69.45 ppm), and C6 (δ 65.46 ppm) [29]. However, the signals identified at δ 96–100 ppm could be attributable to C1 (δ 99.69 ppm), C2 (δ 71.28 ppm), C3 (δ 73.39 ppm), C4 (δ 76.60 ppm), and C5 (δ70.06 ppm) of α-Glc (1→4, 6) residues [30]. The sharp signals at δ 102.59, δ 70.04, and δ 71.28 ppm are attributable to C-1, C-2, and C-3 of the α-Man residues [31]. Based on these results, the monomeric CFPS-1 repeat unit is illustrated in Fig 2C.

**Table 4:** ^1^H and ^13^C NMR chemical shifts for the protein-bound polysaccharide CFPS-1 isolated from *C*. *fluminea* in D_2_O ^a^.

Glycosidic linkage	Chemical shifts (ppm)						
	H-1/C-1	H-2/C-2	H-3/C-3	H-4/C-4	H-5/C-5	H-6/C-6	
→6)-α-D-Glc*p*-(1→	4.89/97.63	3.53/71.31	3.59/73.27	3.34/70.09	3.75/69.45	3.91^b^	65.46
						3.73^c^	
→4, 6)-α-D-Glc*p*-(1→	4.98/99.69	3.54/71.28	3.73/73.39	3.58/76.60	4.03/70.06	3.68^b^	65.83
						3.95^c^	
α-D-Manp-(1→	5.14/102.59	4.09/70.04	3.89/71.28	3.79/69.41	3.80/73.39	3.97^b^	60.49
						3.72^c^	

^a^ The ^1^H NMR and ^13^C NMR spectra were recorded with a 600 MHz Bruker Avance^™^ 600 spectrometer at 60°C. Chemical shifts were reported relative to internal acetone at δH 2.23 ppm for ^1^H spectrum and δC 32.45 ppm for ^13^C spectrum.

^b^ Chemical shift for H-6a.

^c^ Chemical shift for H-6b.

### Antiproliferation and Cytotoxicity Assays

**Fig 3:**
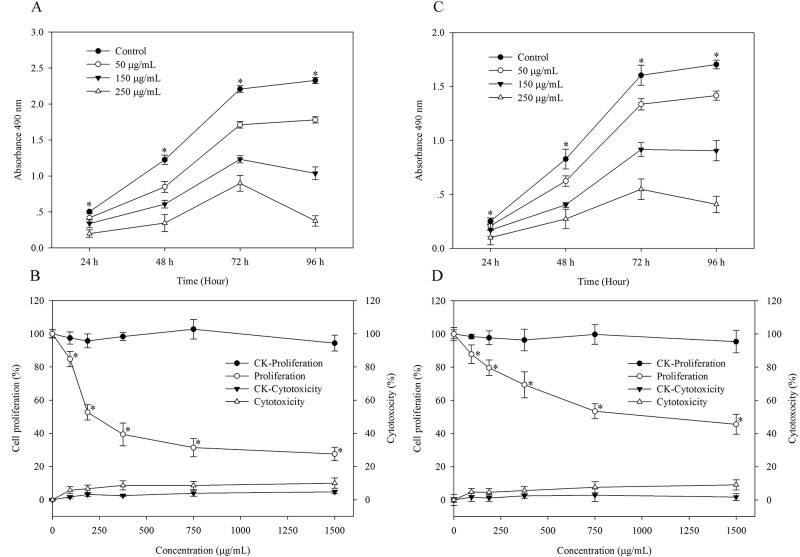
Effect of the *C*. *fluminea* protein-bound polysaccharide CFPS-1 on growth of human breast cancer MCF-7 and MDA-MB-231 cells. The inhibitory effect of CFPS-1 on the cell growth of MCF-7 (A) and MDA-MB-231(C) cells at 24, 48, 72 and 96 h. Proliferation and cytotoxicity against MCF-7 (B) and MDA-MB-231 (D) cells by the CFPS-1 at 72 h. CK, Control of proliferation or cytotoxicity. All data were expressed as mean ± SD of three experiments and each experiment included triplicate repeats. Values marked with * are significantly different from the control (*p* < 0.05).

The antiproliferative effects of CFPS-1 on both MCF-7 and MDA-MB-231 cells were determined with the method developed in our laboratory [20, 21]. As shown in Fig 3(A) and 3(C), CFPS-1 (≥50 μg/mL) dose- and time-dependently inhibited the proliferation of both MCF-7 and MDA-MB-231 cells (Figure A in S1 File). However, the MCF-7 cells were more sensitive to CFPS-1 than the MDA-MB-231 cells. With 250 μg/mL CFPS-1, the proliferation of the MDA-MB-231 and MCF-7 cells was inhibited by 38% and 64%, respectively, after 72 h. The amount of CFPS-1 required to inhibit the proliferation of MDA-MB-231cells by 50% (IC_50_) was 1142 ± 14.84 μg/mL, whereas CFPS-1 more effectively inhibited MCF-7 cell proliferation, with an IC_50_ value of 243 ± 6.79 μg/mL. To further evaluate the antiproliferative and cytotoxic effects of CFPS-1 on human breast cancer MCF-7 and MDA-MB-231 cells, the dose of CFPS-1 was increased from 250 to 1500 μg/mL. At concentrations > 250 μg/mL, CFPS-1 also dose-dependently inhibited MCF-7 and MDA-MB-231 cell proliferation. At the highest dose (1500 μg/mL), MCF-7 and MDA-MB-231 cell proliferation was inhibited by about 78.8% and 64.2%, respectively. CFPS-1 was not cytotoxic to the MCF-7 and MDA-MB-231 cell lines at ≤ 250 μg/mL, as shown in Fig 3(B) and 3(D). Therefore, the low doses of CFPS-1 (from 50 to 250 μg/mL) were chosen for the subsequent experiments.

### Effects of CFPS-1 on the Cell-cycle Distribution in MCF-7 and MDA-MB-231 Cells

**Fig 4:**
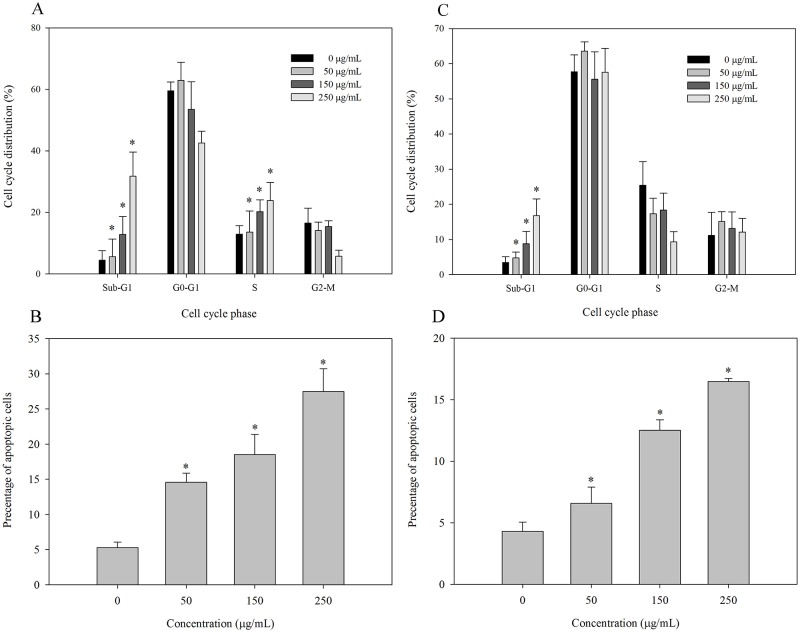
Effect of the *C*. *fluminea* protein-bound polysaccharide CFPS-1 on MCF-7 and MDA-MB-231 cell cycle progression. Cells were cultured for 24 h with CFPS-1 (0, 50, 150 and 250 μg/mL, respectively). Untreated cells (0 μg/mL) were used as control. Cell cycle distribution (%) of MCF-7 (A) and MDA-MB-231 (C) cells were determined by flow cytometry after CFPS-1 treatment; Apoptotic rate (%) of MCF-7 (B) and MDA-MB-231 (D) cells treated with CFPS-1. Apoptotic cells were identified with a TUNEL technique and counted with a light microscope (magnification, ×40). All data were expressed as mean ± SD of three experiments and each experiment included triplicate repeats. Values marked with * are significantly different from the control (*p* < 0.05).

The capacity of CFPS-1 to inhibit the progression of the cell cycle was evaluated with flow cytometry (Fig 4 and Figure B in S1 File). The sub-G1 cell fraction was considered to represent apoptotic cells. Fig 4A shows that CFPS-1 produced 1.7-, 4.3-, and 9.7-fold increases in the apoptosis of MCF-7 cells at concentrations of 50, 150, and 250 μg/mL, respectively, compared with that induced with 0 μg/mL CFPS-1 (Control). The effects of CFPS-1 treatment for 24 h on the MCF-7 cell-cycle phase distribution are also shown in Fig 4B. Compared with the control (17.8%), treatment with 50, 150, or 250 μg/mL CFPS-1 for 24 h increased the population of cells in S phase to 19.3%, 21.2%, or 26.7%, respectively. These results show that CFPS-1 treatment of MCF-7 cells induced the accumulation of S phase cells. To prove the increasing of S phase was not caused by the accelerated DNA synthesis, the effects of CFPS-1 on DNA synthesis was assessed by BrdU assay. The result shows that 50 μg/mL CFPS-1 decreased DNA synthesis compared with the control (0 μg/mL), and was much smaller than the effect of 100 μg/mL CFPS-1 (Figure C in S1 File). Fig 4B also shows that as the concentration of CFPS-1 increased, the sub-G1 apoptotic fraction of cells also increased significantly from 2.5% to 26.8%. Therefore, the induction of S-phase arrest and an increased sub-G1 apoptotic fraction may be the major mechanisms by which the growth of MCF-7 cells is inhibited. In contrast, in MDA-MB-231, with limited change of cells in G0/G1, S, and G2/M phase, CFPS-1 increases the proportion of cells in apoptotic sub-G1 phase (data not shown).

### CFPS-1 Induces Apoptosis of MCF-7 and MDA-MB-231 Cells *In Vitro*

To confirm that exposure to CFPS-1 causes the death of MCF-7 and MDA-MB-231 cells by apoptosis *in vitro*, a TUNEL assay was performed. MCF-7 and MDA-MB-231 cells were treated with different concentrations of CFPS-1 (0, 50, 150, or 250 μg/mL), and then stained with methyl green (Figure D in S1 File). As shown in Fig 4B and 4D, the percentage of apoptotic MCF-7 and MDA-MB-231 cells was counted under light microscopy (magnification, ×40). Treatment with CFPS-1 increased the apoptotic staining in a dose-dependent manner in MCF-7 cells (5.3 ± 2.1% in the control, 15 ± 3.4% in the 50 μg/mL group, 21 ± 5.2% in the 150 μg/mL group, 37 ± 7.8% in the 250 μg/mL group) and MDA-MB-231 cells (4.8 ± 0.4% in the control, 7.2 ± 1.4% in the 50 μg/mL group, 12.2 ± 2.2% in the 150 μg/mL group, 17.3 ± 0.8% in the 250 μg/mL group). These data corroborate the CFPS-1-induced sub-G1 apoptotic fractions determined with flow cytometry shown above.

### Mechanisms of Cell Cycle Arrest and Apoptosis after CFPS-1 Treatment

**Fig 5:**
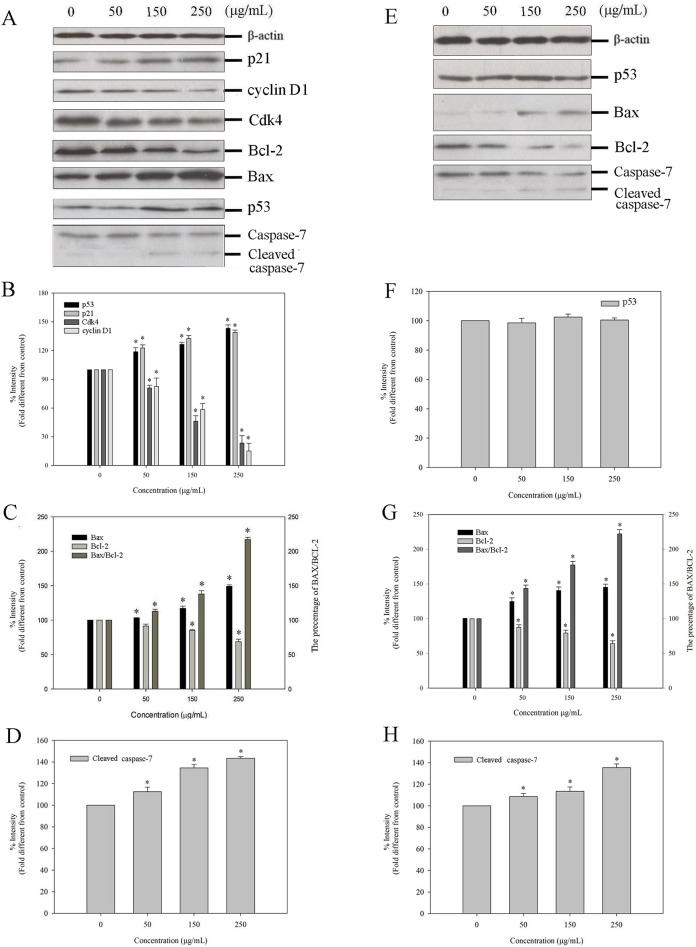
Effects of the *C*. *fluminea* protein-bound polysaccharide CFPS-1 on the expression of p21, Cyclin D1, Cdk4, p53, Bcl-2, Bax and caspase-7 proteins associated with cell cycle and apoptosis in MCF-7(A, B, C and D) and MDA-MB-231 (E, F, G and H) cells. The relative expression of protein was quantified densitometrically (%). Western blot analysis was performed in triplicate per experimental point; β-actin was used as reference control. Values marked with * are significantly different from the control (*p* < 0.05).

To clarify the mechanism of CFPS-1-induced cell-cycle arrest, the expression of p53, p21, Cdk4 and cyclin D1 were evaluated with western blotting, as shown in Fig 5. β-Actin was used as the internal control. The treatment of MCF-7 cells with CFPS-1 dose-dependently increased the levels of p53 and p21 (Fig 5B). In contrast, cyclin D1 and Cdk4 levels were dose-dependently reduced (Fig 5B). But in MDA-MB-231, no obvious accumulation and activation of p53 were observed (Fig 5F). To assess the potential signaling pathways involved in CFPS-1- induced apoptosis; we evaluated the expression of the Bcl-2 family proteins. As shown in Fig 5C and 5G, treatment with CFPS-1 could up-regulated the expression of Bax but down-regulated the expression of Bcl-2, and both effects were dose-dependent. The Bax/Bcl-2 ratio increased approximately 2.43 and 2.27-fold at concentration of 250 μg/mL for MCF-7 and MDA-MB-231 cells, respectively, relative to that of the control. For evaluation of the executor pathway of CFPS-1-induced apoptosis, caspase-7 and cleaved caspase-7 were also measured by western blot. Activation of caspase-7 was significantly increased in both MCF-7 and MDA-MB-231 cells after CFPS-1 treatment, resulting in increased expression of cleaved caspase-7 and decreased expression of caspase-7 (Fig 5D and 5H).

## Discussion

Breast cancer is widely recognized as the most common malignancy in women throughout the world [32,33]. Natural polysaccharide compounds have attracted increasing interest as nontoxic chemopreventive agents capable of inducing tumor cell death in various cancer cell lines [10]. However, most of these polysaccharides have been isolated from plants or fungi and few have been derived from mollusks. In our previous study, two water-soluble polysaccharides (CFPS-2 and CFPS-1) were isolated and purified from *C*. *fluminea*, a freshwater bivalve mollusk. We also found that CFPS-2, a sulfated polysaccharide, with an average Mw of about 22 kDa, was shown to significantly inhibit the proliferation of several cancer cell lines *in vitro* [2].

But few researches on CFPS-1 were reported because of its complex chemical structure. In this study, the chemical characteristics and inhibitory effects of CFPS-1 on human breast cancer cells were further assessed. This is the first study to show that the *C*. *fluminea* polysaccharide CFPS-1, a novel polysaccharide–protein complex, with an average Mw of about 283 kDa, significantly and dose-dependently inhibits the growth of human breast cancer MCF-7 and MDA-MB-231 cells *in vitro*. And the possible mechanisms were also demonstrated.

The suppression of oncogenesis often involves the regulation of signal transduction pathways, resulting in cell growth arrest and then apoptosis [34]. Cell-cycle progression is decelerated by cyclin-dependent kinases (CDKs) inhibitors (such as p27, p21, and p16) and p53, and accelerated by CDKs and cyclins. It is also well known that p53, which is closely associated with cancer inhibition, plays vital roles in a variety of intracellular and extracellular regulatory mechanisms, exerting its functions predominantly through the transcriptional activation of target proteins, such as Cdk 4, the Cdk inhibitor p21, and cyclin D1, to induce cell-cycle arrest, and the pro-apoptotic protein Bax to induce apoptosis [35]. The results of the present study suggest that CFPS-1 blocks the growth of MCF-7 cells by arresting them in the S phase of the cell cycle. Further analysis suggested that treating MCF-7 cells with CFPS-1 significantly up-regulated the expression of p53 and p21 and down-regulated that of cyclin D1 and Cdk4. Therefore, p53 may be one of the upstream regulators of cell-cycle arrest in CFPS-1-treated MCF-7 cells. However, in MDA-MB-231 cells, p53 is in mutant form and was dysfunctional. Thus, no obvious accumulation and activation of p53 were observed. This result is in agreement with a previous investigation [36].

Apoptosis, a process of programmed cell death, is triggered by a variety of physiological conditions. This type of cell death is regulated by many gene products that promote or block cell death at different stages. In addition to p53, there is mounting evidence that the ratios of pro- (Bax and Bid) and anti-apoptotic (Bcl-2 and Bcl-xL) Bcl-2 family proteins, including the Bcl-2/Bax ratio rather than Bcl-2 alone, are important in the regulation of apoptosis [37–39]. Moreover, caspases are a family of cysteine proteases that play a central role during the executional phase of apoptosis [40]. Our study demonstrates that treatment of MCF-7 and MDA-MB-231 cells with CFPS-1 significantly increases the expression of Bax and cleaved caspase-7 but reduces that of Bcl-2 and caspase-7, suggesting that CFPS-1 increase caspase activities through the ratios of pro- and anti-apoptotic Bcl-2 family proteins and these occurrences of mitochondrial apoptotic events play an important role in CFPS-1-mediated apoptosis.

The anticancer effects of polysaccharides are strongly related to their chemical structures. A previous study of human breast cancer showed that polysaccharides that contain β-glucan structures, such as β-1,3-glucan or β-1,6-glucan, have important anticancer activities [41]. However, some polysaccharides with different structures, such as α-glucan, also play important roles in the growth inhibition of several cancer cell lines [10]. As far as we know, there have been few reports of polysaccharides with structures similar to that of CFPS-1, a protein-bound polysaccharide mainly linked by α-configuration glycosidic bonds with a mean *M*w of about 283 kDa, inducing both cell growth arrest and apoptosis in human breast tumor (MCF-7 and MDA-MB-231) cells. In the peptide moiety, fifteen amino acids in the CFPS-1 were also detected, and were consistent with a previous study showing that a peptide from *Elysia rufescens*, a marine gastropod mollusk, significantly *in vitro* and *in vivo* suppressed cellular proliferation in human breast tumor cell lines [42]. Therefore, this study provides novel insight into the structural basis of a protein-bound polysaccharide with anticancer effects *in vitro* and its mechanism.

The limitation of this study is that the data obtained here may not completely represent the *in vivo* situation. CFPS-1, which is known as a high molecule weight (283 kDa) polysaccharide, might not be applied intravenously and would be digested in the gastrointestinal tract after oral ingestion in animal model. So how did the orally ingested polysaccharide come into or contact with breast cancer cells, and be used for prevention or treatment of breast cancer *in vivo*? In previous studies, some of the functional domains in high molecular polymer can be absorbed through the intestinal tract and exhibit bioactivities [43–44]. Therefore, investigation on CFPS-1 functional domains including the anti-cancer effect by oral administration of the CFPS-1 and animal studies *in vivo* is an area that still requires further research.

In conclusion, a novel protein-bound polysaccharide, designated CFPS-1, was isolated and purified from *C*. *fluminea*. The glycoprotein is mainly composed of mannose and glucose in a molar ratio of 3.1: 12.7, with a mean *M*w of about 283 kDa. The glycosyl residues of CFPS-1 are mainly linked by α-configuration glycosidic bonds, and the repeating unit of the structure has been described above. Preliminary bioactivity tests conducted *in vitro* revealed that CFPS-1 effectively inhibits the growth of MCF-7 and MDA-MB-231 cells. In MCF-7 cells, CFPS-1 produced a significant up-regulation of p53, p21, Bax and cleaved caspase-7 but down-regulation of Cdk4, cyclin D1, Bcl-2 and caspase-7. These effects resulted in cell cycle blockade at the S-phase and apoptosis induction. In contrast, in MDA-MB-231, with limited degree of change in cell cycle distribution, CFPS-1 increases the proportion of cells in apoptotic sub-G1 phase executed by down-regulation of Bcl-2 and caspase-3 and up-regulation of Bax and cleaved caspase-3. These findings may aid in the understanding of the mode of tumor-inhibitory action of CFPS-1 and provide a theoretical basis for the prevention or treatment of human breast cancer with *Corbicula fluminea* polysaccharide extracts.

## Supporting Information

S1 File This file contains all Supporting Figures (A-D). **Figure A.** Standard curves determined by MTS assay. This information is used for estimation of cell numbers. **Figure B.** Effect of the *C*. *fluminea* protein-bound polysaccharide CFPS-1 on MCF-7 cell cycle progression. This figure corresponds to Fig 4A. **Figure C.** Effect of CFPS-1 on DNA synthesis. The result shows that CFPS-1 decreased DNA synthesis. **Figure D.** TUNEL staining of MCF-7 cells with or without polysaccharide. This figure corresponds to Fig 4B.
(DOC)

S2 File This file contains the raw data used in the paper. (XLSX)
