# Supplementary material for: Protein-Bound Polysaccharide from Corbicula fluminea Inhibits Cell Growth in MCF-7 and MDA-MB-231 Human Breast Cancer Cells
Source: PLoS One. 2016 Dec 13;11(12):e0167889. doi: 10.1371/journal.pone.0167889 (PMC5154514; doi:10.1371/journal.pone.0167889)
Supplement: S1 File — Figure A. Standard curves determined by MTS assay. This information is used for estimation of cell numbers. Figure B. Effect of the C. fluminea protein-bound polysaccharide CFPS-1 on MCF-7 cell cycle progression. This figure corresponds to Fig 4A. Figure C. Effect of CFPS-1 on DNA synthesis. The result shows that CFPS-1 decreased DNA synthesis. Figure D. TUNEL staining of MCF-7 cells with or without polysaccharide. This figure corresponds to Fig 4B. (DOC) [file pone.0167889.s001.doc]

Supporting Information for

**Protein-bound Polysaccharide from *Corbicula fluminea*** **Inhibits Cell Growth in MCF-7 and MDA-MB-231 Human Breast Cancer Cells**

Ningbo Liao^1^, Jianjun Zhong^2^, Ronghua Zhang^1^, Xingqian Ye^2^, Yanjun Zhang^1^, Wenjun Wang^2^, Yuexia Wang^4^, Shiguo Chen^2^, Donghong Liu^2,5*^and Ruihai Liu^3*^

^1^ Department of Nutrition and Food Safety, Zhejiang Provincial Center for Disease Control and Prevention, Hangzhou, Zhejiang, China

^2^ College of Biosystem Engineering and Food Science, Zhejiang University, Hangzhou, Zhejiang, China

^3^ Department of Food Science, Cornell University, Ithaca, New York, United State

^4^ Zhejiang Academy of Science & Technology for Inspection & Quarantine, Hangzhou, Zhejiang, China

^5^ Fuli Institute of Food Science, Zhejiang University, Hangzhou, Zhejiang, China

* Corresponding author

E-mail: [dhliu@zju.edu.cn](mailto:dhliu@zju.edu.cn) (D. Liu); rl23@cornell.edu (R. Liu)

**Figure A.** Standard curves determined by MTS assay


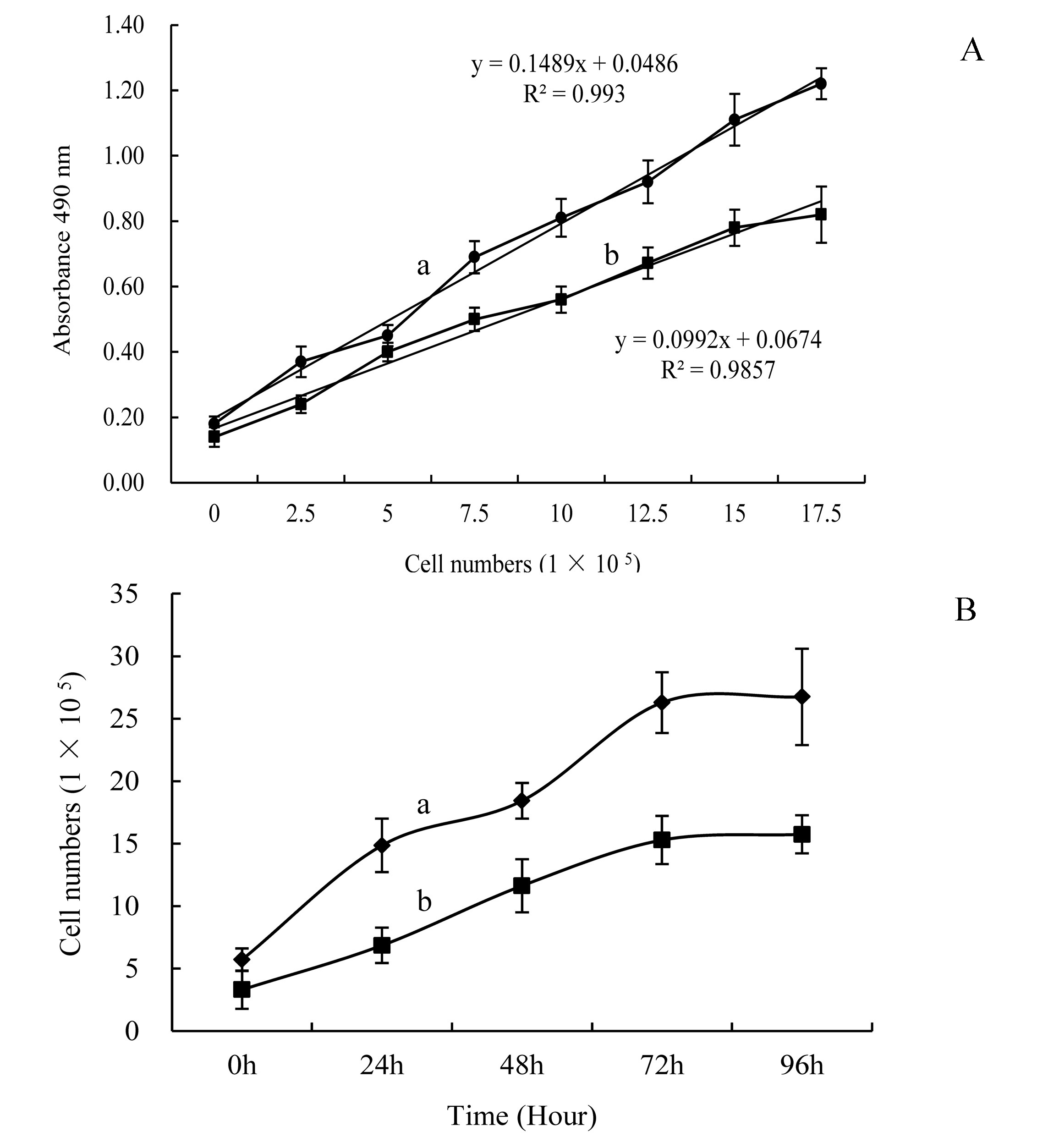


A: Relationships of cell numbers to the relative absorbance of MCF-7 (a) and MDA-MB-231 (b); B: Cell growth curves determined by MTS assay in MCF-7 cells (a) and MDA-MB-231 cells (b) *in vitro*. All data were expressed as mean ± SD of three experiments and each experiment included triplicate repeats.

**Figure B.** Effect of the *C*. *fluminea* protein-bound polysaccharide CFPS-1 on MCF-7 cell cycle progression. Cells were cultured for 24 h with CFPS-1 (A=0, B=50, C=150 and D=250 μg/mL, respectively). Untreated cells (0 μg/mL) were used as control.

**
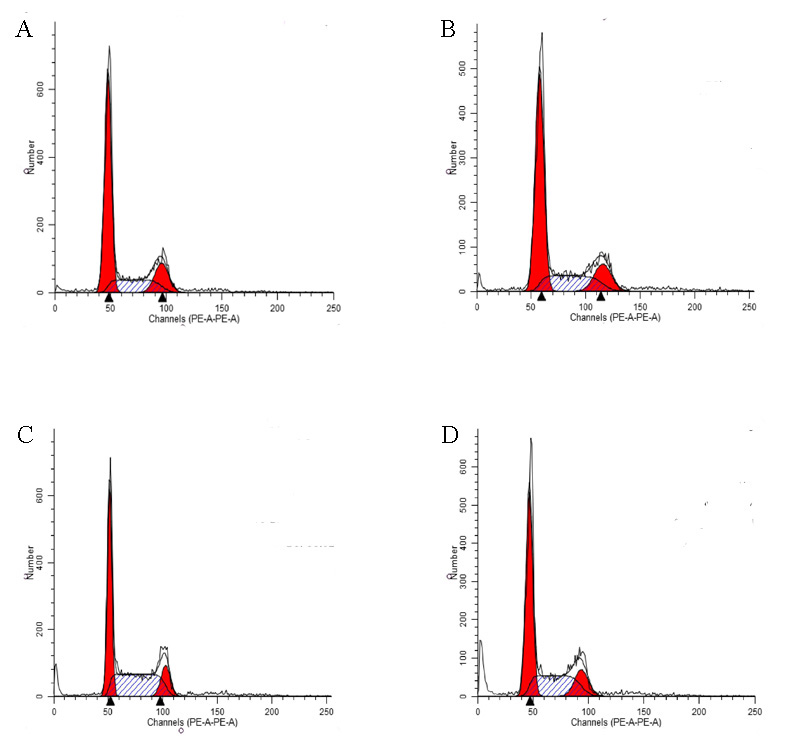
**

Cell cycle was analyzed with flow cytometry. One representative of three repeat experiments was shown here.

**Figure C.** Effect of CFPS-1 on DNA synthesis


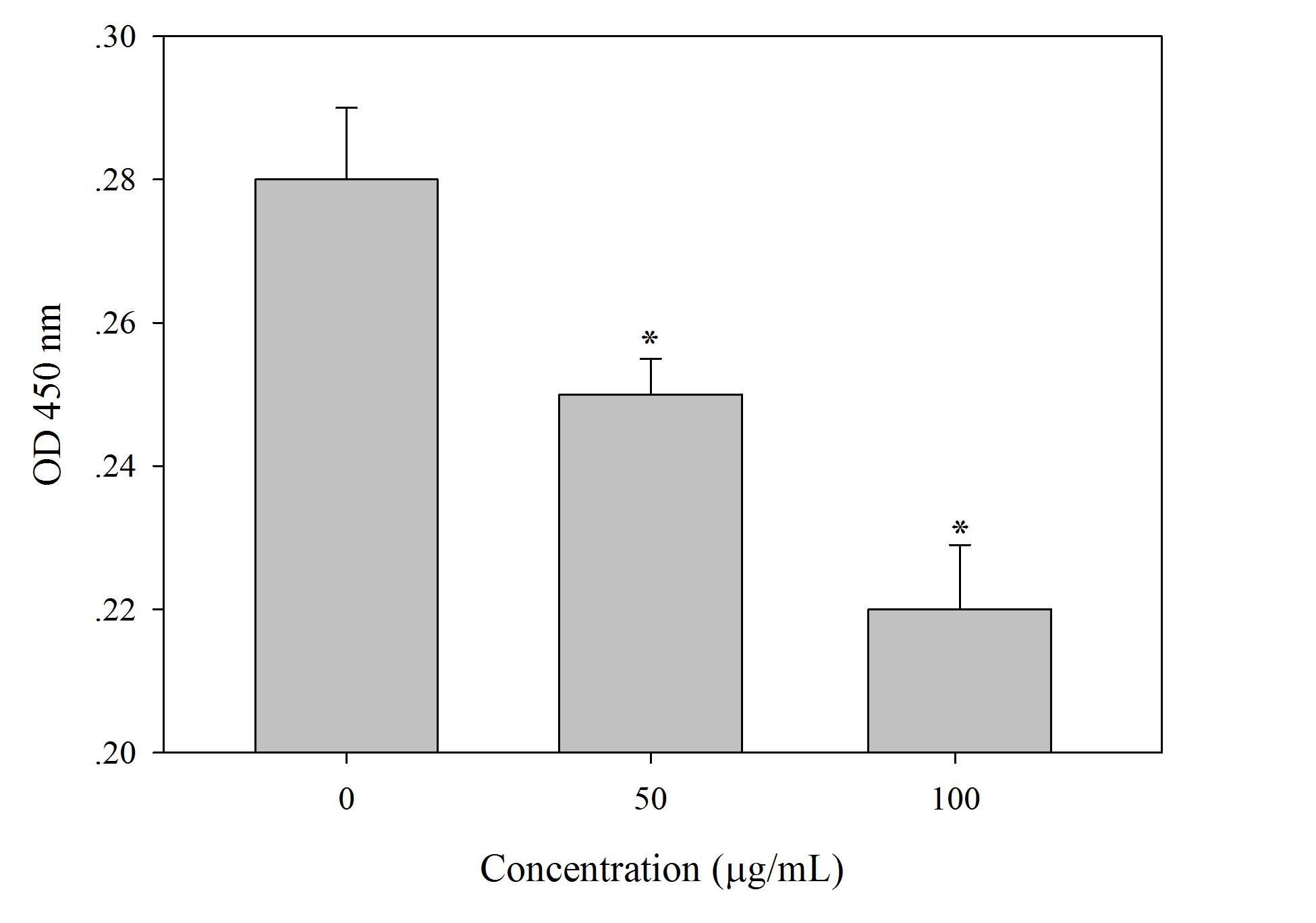


MCF-7 cells were treated with 0, 50 and 100 μg/mL CFPS-1, and DNA synthesis was measured by BrdU assay at 72 h. All data were expressed as mean ± SD of three experiments and each experiment included triplicate repeats. Values marked with * are significantly different from the control (*p* < 0.05).

**Figure D.** TUNEL staining of MCF-7 cells with or without polysaccharide.


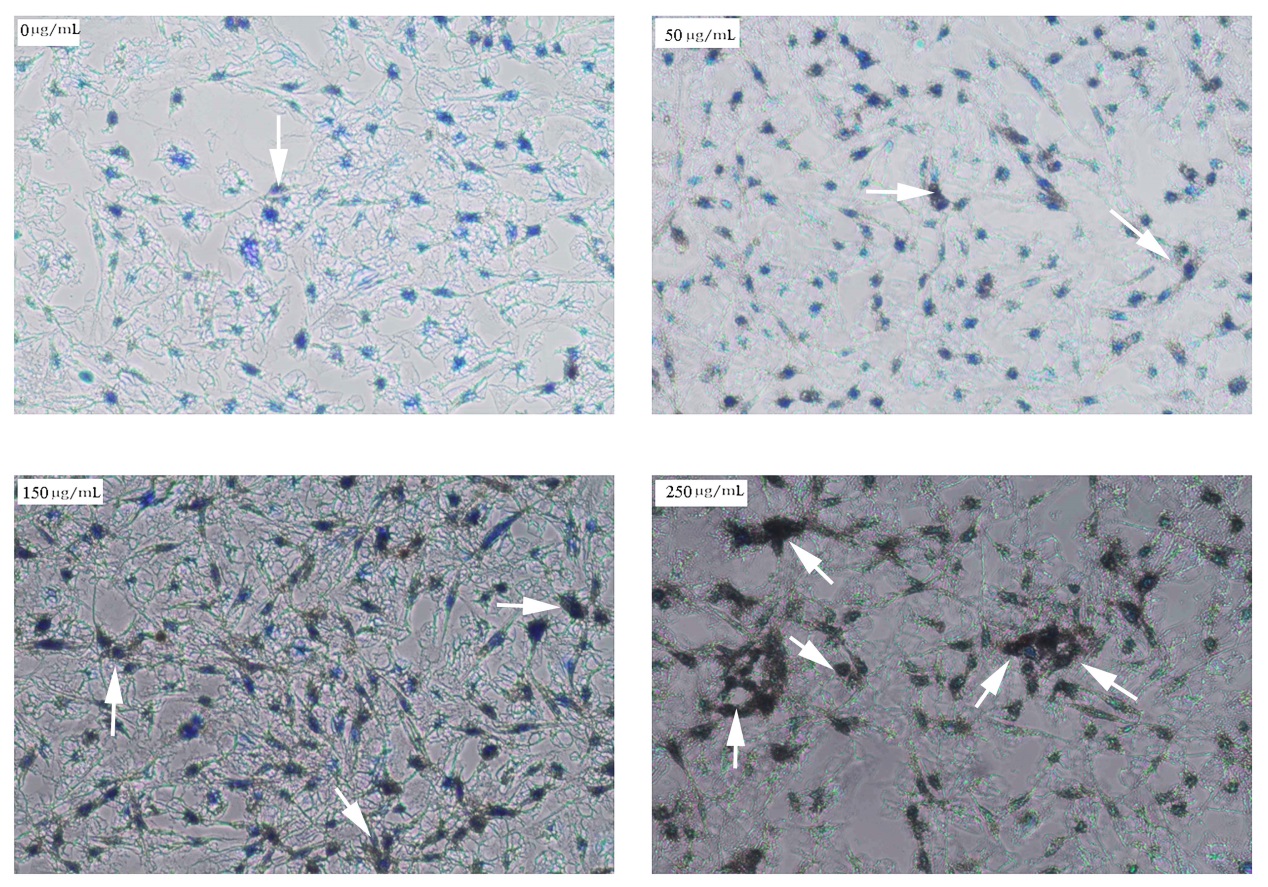


Cells were cultured for 24 h with polysaccharide: 50, 150 and 250 μg/mL. Untreated cells (0 μg/mL) were used as control. After treatment, cells were isolated and prepared for measurement of TUNEL staining using light microscopy (magnification, × 40). Arrows indicate apoptotic cells. Figures are representative of three separate experiments.
